# Structure of the nuclease subunit of human mitochondrial RNase P

**DOI:** 10.1093/nar/gkv481

**Published:** 2015-05-07

**Authors:** Linda Reinhard, Sagar Sridhara, B. Martin Hällberg

**Affiliations:** 1 Department of Cell and Molecular Biology, Karolinska Institutet, 17177 Stockholm, Sweden; 2 Röntgen-Ångström-Cluster, Karolinska Institutet Outstation, Centre for Structural Systems Biology, DESY-Campus, 22607 Hamburg, Germany; 3 European Molecular Biology Laboratory, Hamburg Unit, 22603 Hamburg, Germany

## Abstract

Mitochondrial RNA polymerase produces long polycistronic precursors that contain the mRNAs, rRNAs and tRNAs needed for mitochondrial translation. Mitochondrial RNase P (mt-RNase P) initiates the maturation of the precursors by cleaving at the 5′ ends of the tRNAs. Human mt-RNase P is only active as a tripartite complex (mitochondrial RNase P proteins 1–3; MRPP1-3), whereas plant and trypanosomal RNase Ps (PRORPs)—albeit homologous to MRPP3—are active as single proteins. The reason for this discrepancy has so far remained obscure. Here, we present the crystal structure of human MRPP3, which features a remarkably distorted and hence non-productive active site that we propose will switch to a fully productive state only upon association with MRPP1, MRPP2 and pre-tRNA substrate. We suggest a mechanism in which MRPP1 and MRPP2 both deliver the pre-tRNA substrate and activate MRPP3 through an induced-fit process.

## INTRODUCTION

Transcription in human mitochondria gives rise to two long polycistronic transcripts that are subsequently processed into mature mRNAs, tRNAs and rRNAs by the mitochondrial RNA-processing machinery. According to the tRNA punctuation model (1), the tRNAs are processed first, and recent work indicates that the primary processing is performed co-transcriptionally through tRNA 5′-processing by mitochondrial RNase P (mt-RNase P) (2–5). In humans and probably most other metazoans, mt-RNase P is composed of three protein subunits (mitochondrial RNase P proteins 1–3; MRPP1-3) (6). MRPP1 (also known as TRM10C: tRNA methyltransferase 10 homolog) and MRPP2 (also known as SDR5C1: short chain dehydrogenase/reductase family 5C member 1) form a strong protein complex (6) and perform the *N^1^*-methylation of adenosine and guanosine at position 9 (m^1^A9 and m^1^G9, respectively) of human mt-tRNAs (7–9). This methylation is necessary for the proper folding of at least one mitochondrial tRNA (8); in fact, a majority of human mitochondrial tRNAs contain an m^1^A9 or m^1^G9 (10). The methyltransferase activity is located in MRPP1, while the exact role of MRPP2 is unclear (9). In addition, the methyltransferase activity of MRPP1/2 and the endonuclease activity of mt-RNase P are functionally independent (9). The third subunit, MRPP3 (also known as PRORP: proteinaceous RNase P), contains the sequence features of a zinc-binding domain and an N-terminal, putative RNA-binding pentatricopeptide repeat (PPR) domain, where the PPR repeats are helix-turn-helix motifs of ∼35 amino acids and are found in a large number of RNA binding eukaryotic proteins (11). Furthermore, the MRPP3 subunit harbors a C-terminal domain that carries the sequence features of a metallonuclease; MRPP3 has therefore been assumed to be the endonucleolytic subunit of mt-RNase P (6,12).

In contrast to human mitochondria, plants and trypanosomes use MRPP3 homologs (e.g. PRORP1-3, in *Arabidopsis thaliana*) that function as single-subunit mt-RNase Ps (13–16). *A. thaliana* PRORP1 localizes to mitochondria as well as chloroplasts, while PRORP2 and PRORP3 are found in the nucleus (13,15). PRORP1 has 22% sequence identity to MRPP3 and a PRORP1 crystal structure shows a V-shaped tripartite structure with a C-terminal metallonuclease domain of the NYN (N4BL1, YacP-like nuclease) family, with a typical and functional two-metal-ion catalytic site that has conserved aspartate residues (17,18). Furthermore, the PRORP1 PPR domain recognizes the D and TΨC loops in folded precursor tRNAs (19,20). In fact, the active site residues of the PRORP1 are conserved in human MRPP3, thus raising the question as to why human mt-RNase P is a multi-subunit system, whereas plant and trypanosomal RNase P is a single-subunit system. We reasoned that MRPP3 is hindered from being active on its own by a structural latch in some form; to test this hypothesis, we determined the high-resolution crystal structure of human mitochondrial MRPP3.

## MATERIALS AND METHODS

### Construction of expression vectors

Human MRPP3 and different N-terminal truncations of MRPP3 were cloned from the Mammalian Gene Collection (gi:21595090) into the pNIC28-Bsa4 (gi:124015065) vector for expression of N-terminally His_6_-tagged proteins. All MRPP3 mutants are based on mature, full-length MRPP3 lacking the mitochondrial target sequence (residues 45–583). Single and double mutants were generated by site-directed mutagenesis. Longer sequence replacements (for MRPP3-Δlariat, D561 to T574 were replaced by the sequence GSG; for MRPP3-MND^P^, S357 to S544 of MRPP3 were replaced by C355 to P535 of PRORP1) were created by In-Fusion HD cloning (Clontech), using gBlocks (IDT) for the longer replacements. All of the constructs were verified by DNA sequencing.

### Protein expression and purification

All of the MRPP3 variants were produced by following the same protocol. Human MRPP3 and its different variants with an N-terminal His_6_-tag were expressed in *Escherichia coli* strain KRX (Promega) harboring a pRARE2 plasmid (Novagen). The proteins were purified using nickel-affinity and size exclusion chromatography. The N-terminal His_6_-tag was removed by TEV cleavage. The protein, which was in 20 mM HEPES pH 7.5, 300 mM sodium chloride, 10%(v/v) glycerol and 0.5 mM Tris(2-carboxyethyl)phosphine (TCEP), was concentrated by ultrafiltration to 5–25 mg/ml and stored at –80°C. MRPP1 (also: tRNA methyltransferase 10C homolog, TRMT10C) and MRPP2 (also: short chain dehydrogenase/reductase 5C member 1, SDR5C1; or 17β-hydroxysteroid dehydrogenase type 10, HSD10) were produced as described previously (9). After Ni-nitrilotriacetic acid purification, the protein complex was applied to size exclusion chromatography in 20 mM Tris at pH 7.5, 300 mM sodium chloride, 15%(v/v) glycerol and 0.1 mM TCEP.

### Crystallization and data collection

Δ206-MRPP3 (residues 207–583) at a concentration of 25 mg/ml was crystallized using the vapor diffusion method at 298 K. Crystals were obtained in 0.28 M sodium malonate pH 7.0 and 16%(w/v) PEG 3350 (crystal 1); in 30 mM potassium citrate pH 6.0, 16%(w/v) PEG 3350 and 20 mM magnesium chloride (crystal 2); or in 100 mM HEPES pH 8.0, 30%(w/v) PEG 6000 and 20 mM magnesium chloride (crystal 3). Crystal 3 was further soaked with 100 mM magnesium chloride in the reservoir solution. All of the crystals were cryoprotected using the reservoir solution supplemented with 17%(v/v) glycerol and all data collections were performed at 100 K. Anomalous X-ray diffraction data from crystal 1 were collected at beam line P14 at PETRA-III, EMBL/DESY, Germany, using a wavelength of 1.2777 Å. For crystals 2 and 3, X-ray diffraction data were collected at a wavelength of 0.97625 Å at beam line ID29 at ESRF (21), France, and at 0.91841 Å at beam line BL14.1 at BESSY-II (22), Germany, respectively. The X-ray diffraction data (Table 1) were indexed and integrated with XDS (23) and scaled with XSCALE (23). Furthermore, CC1/2 statistics and cutoffs are based on (24).

**Table 1.** Summary of data collection and refinement statistics

	Crystal 1	Crystal 2	Crystal 3
*Crystallization*			
Buffer system	Sodium malonate pH 7.0	Potassium citrate pH 6.0	HEPES pH 7.0
Magnesium concentration		20 mM	crystallization: 20 mM soaking: 100 mM
*Data collection*			
Resolution (Å)	50–2.55 (2.62–2.55)	50–1.8 (1.85–1.80)	50–1.98 (2.03–1.98)
Space group	P4_3_	P4_3_	P4_3_
Cell dimensions *a, b, c* (Å)	99.44, 99.44, 50.98	99.23, 99.23, 50.90	99.04, 99.04, 50.61
Total number of reflections	240837	488725	234939
Number of unique reflections	31756	46231	34479
Completeness (%)	99.9 (100.0)	99.9 (99.8)	99.9 (99.9)
Multiplicity	7.6 (7.5)	10.6 (10.7)	6.8 (7.0)
Mosaicity	0.081	0.075	0.108
*R*_merge_ (%)	8.7 (97.4)	9.2 (227)	9.3 (202.5)
I/σ(I)	15.28 (2.58)	12.89 (0.89)	16.54 (1.0)
CC_1/2_	99.9 (79.3)	99.9 (43.3)	99.9 (47.7)
*Refinement*			
Resolution limits (Å)		50–1.8 (1.89–1.80)	50–1.98 (2.25–1.98)
No. of reflections		46199	34460
*R*/*R*_free_ factor (%)		18.85/22.76	19.29/22.63
*r.m.s.d*.			
Bond lengths (Å)		0.007	0.008
Bond angles (°)		0.920	0.957
*No. atoms*			
Protein		2819	2786
Zn^2+^/glycerol/water		1/12/178	1/12/149
Average B-factor (Å^2^)			
Protein		62.0	64.0
Zn^2+^/glycerol/water		44.0/89.2/50.8	44.3/74.4/50.0
Ramachandran parameters (%)			
Preferred regions		98.77	97.55
Allowed regions		1.23	2.45
Outliers		0	0

The last resolution shell details are given in parentheses. The CC1/2 statistics and cutoffs are based on (24).

### Structure determination and refinement

The zinc ion in the anomalous data set of crystal 1 was located using PHENIX.AUTOSOLVE (25), which automatically built 195 of the 377 expected amino acid residues. Iterative rounds of model building and refinement were carried out manually using COOT (26) and PHENIX.REFINE (25). Reliable model building was not possible for six loop regions of the metallonuclease domain (regions 378–385, 417–421, 450–457, 476–477, 502–509 and 531–534). The geometry of the final model was evaluated using the program MolProbity (27). Figures were prepared using PyMol (www.pymol.org).

### *In-vitro* transcription of pre-tRNAs

The human pre-mt-tRNA was produced *in vitro* using an MS2- and GlmS-based affinity purification setup (28). The DNA template for *Homo sapiens* mitochondrial pre-mt-tRNA^Tyr^ (region 5794–5932) was gene synthesized (IDT). The final pre-mt-tRNA^Tyr^ substrate was 141 ribonucleotides long and contained a 5′-leader of 43 nucleotides, the tRNA sequence of 65 nucleotides and a 3′-trailer of 33 nucleotides.

### *In-vitro* mt-RNase P assay

The mitochondrial RNase P activity was analyzed as described previously (6,9). For all of the cleavage assays, constructs lacking the mitochondrial target sequence (MRPP3 containing residues 45–583 or its mutants) or specific N-terminal truncations were used. The reaction mixture was incubated in assay buffer containing 30 mM 3-(N-morpholino)propansulfonic acid (MOPS) pH 7.6, 150 mM sodium chloride, 5%(v/v) glycerol, 5 mM magnesium chloride, 8 mM S-adenosyl-methionine, 40 nM pre-mt-tRNA^Tyr^, 400 nM MRPP1/2 and 200 nM MRPP3 or its mutants. As a negative control (NC), 100 mM ethylenediaminetetraacetic acid (EDTA) were added to the reaction mix prior to the start of the cleavage reaction. The reactions were incubated at 30°C for 1.5 h and then quenched with 100 mM EDTA. The reaction products were resolved on 7M urea 8% polyacrylamide gels in TBE buffer. After electrophoresis, the reaction products were visualized by staining with SYBR Green II (Invitrogen).

## RESULTS

### Overall structure

**Figure 1.**
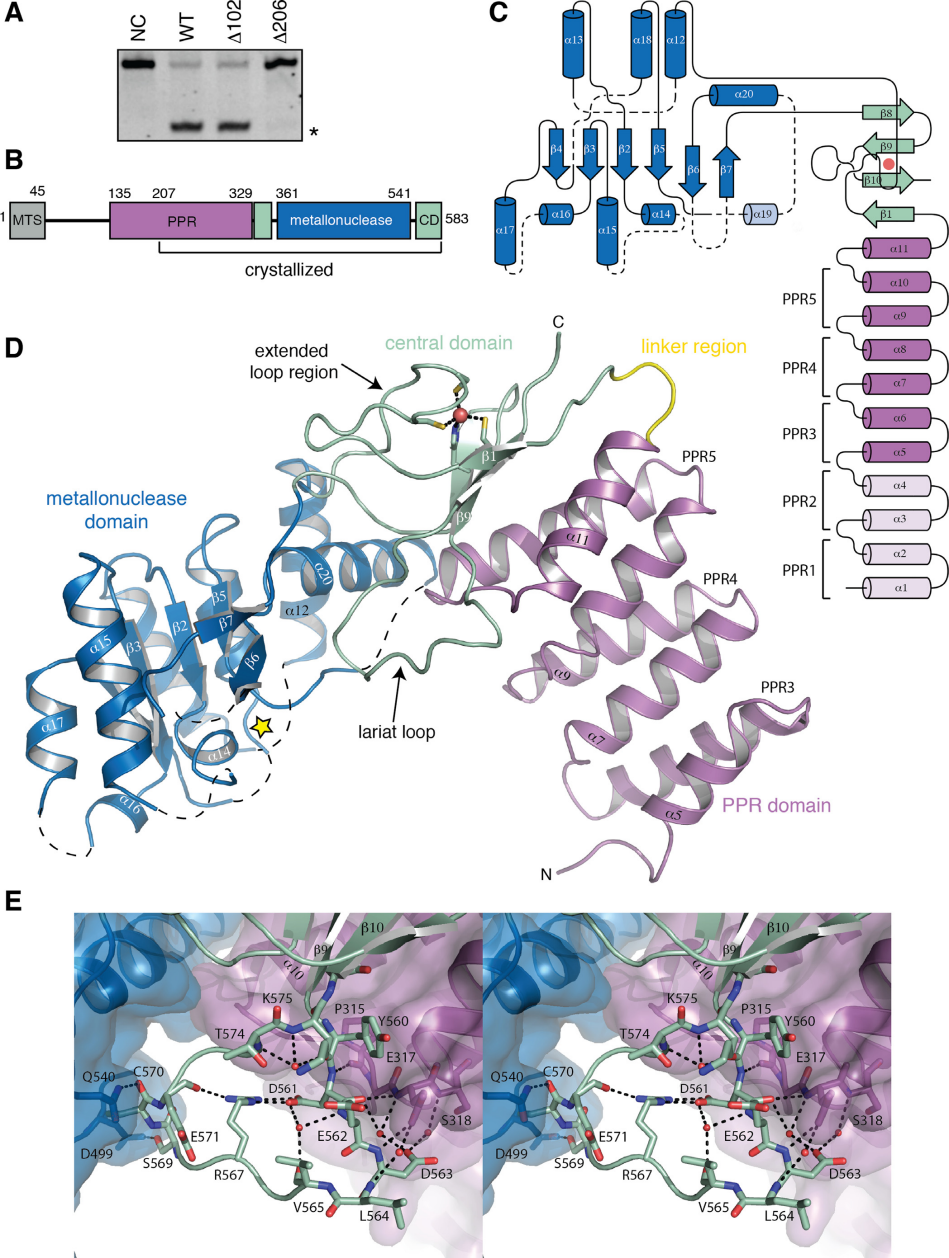
Domain architecture and structure of human MRPP3. (**A**) mt-RNase P assay of three N-terminal MRPP3 truncates. Reactions contain 40 nM pre-mt-tRNA^Tyr^, 400 nM MRPP1/2 and 200 nM MRPP3 or its variants and were resolved on 7M urea 8% polyacrylamide gels. NC is the negative control. The cleavage product is indicated with an asterisk. See Supplementary Figure S1 for an image showing additionally the cleaved off 5′-leader. (**B**) Bar diagram of human MRPP3. Mitochondrial target sequence, MTS; pentatricopeptide repeat domain, PPR; central domain, CD. (**C**) Topology diagram of human MRPP3. The structural elements not visible in the structure are shown in pale colors and dashed lines. The zinc ion is shown by a red sphere. (**D**) Overall fold of Δ206-MRPP3 (1.80 Å resolution). The PPR domain (purple) and metallonuclease domain (blue) are linked by the central domain (green). The short linker region is colored in yellow. The zinc (red sphere) coordinating residues are shown with sticks. A yellow star marks the location of the active site. The first α-helix of the model is assigned as α5. (**E**) Hydrogen bond network in the Δ206-MRPP3 lariat loop. The water molecules are shown in red spheres and interacting residues are shown using sticks.

Mature, full-length human MRPP3 (residues 45–583) persistently resisted crystallization. Activity assays showed that MRPP3 lacking part of the N-terminal flexible region (Δ102-MRPP3; residues 103–583) was fully active, while removal of the N-terminal flexible region and of PPR motif 1–2 (Δ206-MRPP3; residues 207–583) gives raise to inactive enzyme (Figure 1A and B; Supplementary Figure S1). Only this inactive construct produced crystals that diffracted to high resolution, which therefore allowed crystal-structure determination.

Δ206-MRPP3 is an L-shaped molecule composed of an N-terminal PPR domain, a central domain and a metallonuclease domain (Figure 1B–D). The central domain links the two ‘arms’ formed by the PPR and metallonuclease domains, each 35 and 40 Å long, respectively. The N-terminal PPR domain (residues 207–329) contains seven α-helices (α5–α11) that form three PPR motifs (PPR3–5). This is then followed by one additional α-helix (the PPR domain terminating helix, α11), altogether generating a right-handed superhelical structure (Figure 1D).

The PPR domain is connected to the metallonuclease domain through the central domain (residues 334–356 and 545–583), which comprises a four-stranded, anti-parallel β-sheet (β1 and β8–10) and harbors the conserved zinc-binding site. The zinc-binding site is flanked on one side by an extended loop region that succeeds strand β1 (Figure 1D), while the opposite side of the β-sheet packs against the PPR domain through interactions with α11 and PPR5. A long loop region between β9 and β10 (hereafter referred to as the *lariat* loop; residues 560–575) intercalates between the metallonuclease and PPR domains. The lariat loop winds through a network of water-mediated hydrogen bonds (Figure 1E). Specifically, D561, D563 and L564 in the lariat loop interact through direct and water-mediated hydrogen bonds with residues of the loop region that connect PPR5–α10 and α11. The lariat loop conformation is additionally stabilized by a salt bridge between D561 and R567 (Figure 1E). Furthermore, the S569 hydroxyl group and C570 backbone carbonyl group in the lariat loop interact intimately with the presumed metallonuclease active-site region.

The overall structure of the metallonuclease domain (residues 361–541) belongs to the NYN family (18) and comprises a central, four-stranded, parallel β-sheet (β2-β7) that is flanked on both sides by α-helices (Figure 1D). The loop connections on the side of the β-sheet that faces away from the presumed tRNA-binding interface appear well ordered, whereas the segments connecting secondary structure elements at the binding interface are disordered with non-interpretable electron density, probably due to pronounced flexibility.

### The active site of MRPP3 is distorted in the absence of MRPP1/2 and pre-tRNA

**Figure 2.**
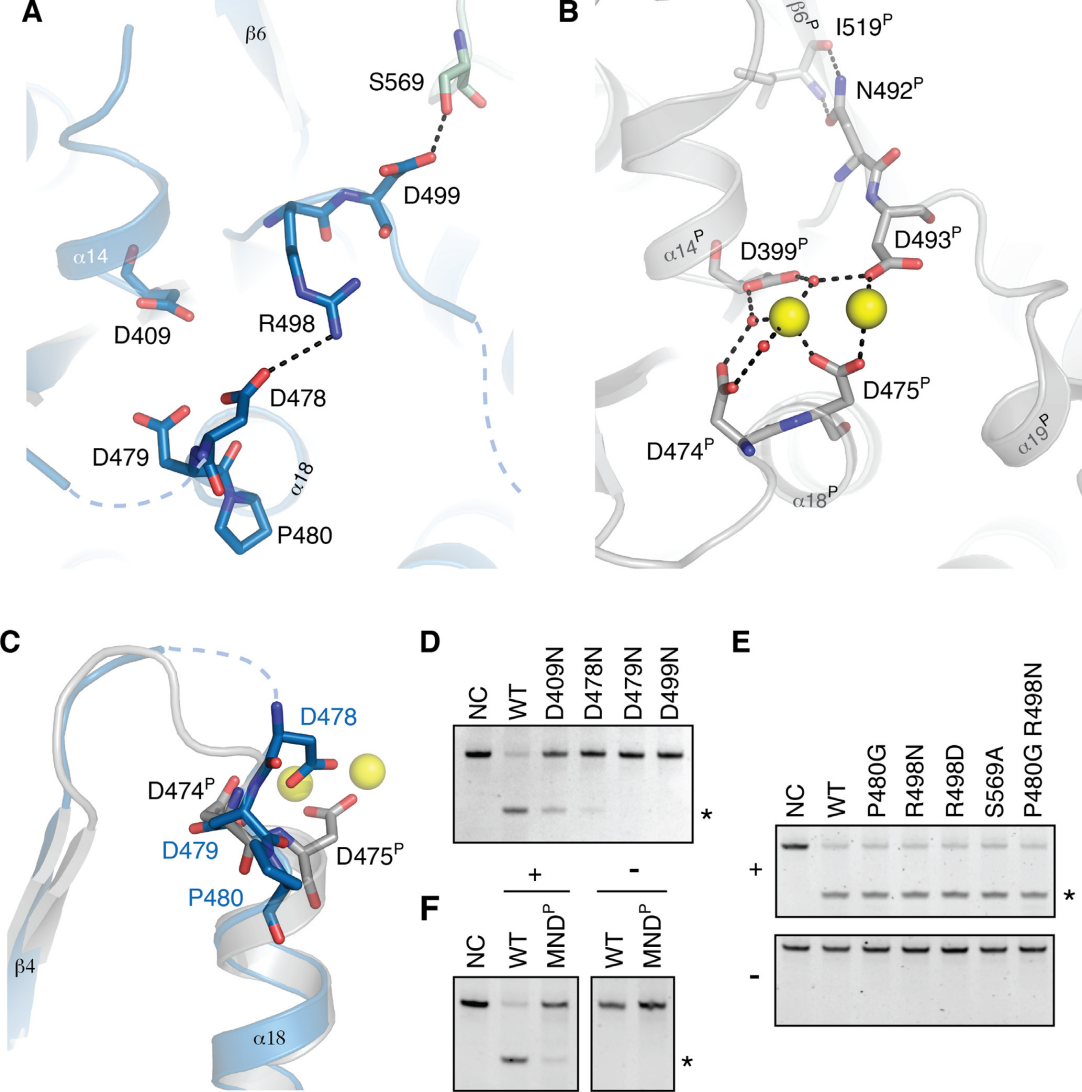
The active site of Δ206-MRPP3. (**A**) Active-site of human MRPP3 (1.80 Å resolution). Functionally important residues are shown using sticks. (**B**) Active site of *A. thaliana* PRORP1 in complex with Mn^2+^ (yellow spheres; PDB entry 4G24, 1.95 Å resolution, (17)). The orientation of the active site is the same as in A. (**C**) Arrangement of the ‘swapped’ aspartate residues. Following the backbone trace, MRPP3 D478 and D479 (in blue) swap positions when compared to PRORP1 D474^P^ and D475^P^ (in gray). (**D**) mt-RNase P assay of active-site mutants. (**E**) mt-RNase P assay of mutations of MRRP3 aimed at obtaining MRPP1/2-independent activity. Reactions contain 40 nM pre-mt-tRNA^Tyr^, 400 nM MRPP1/2 (if applicable) and 200 nM MRPP3 or its mutants and were resolved on 8% denaturing PAGE. The activity was assayed in the presence (+) and absence (−) of MRPP1/2. NC is the negative control. (**F**) mt-RNase P activity of MRPP3 exhibiting the metallonuclease domain of *A. thaliana* PRORP1 (MRPP3-MND^P^) assayed in the presence (+) and absence (−) of MRPP1/2.

Based on sequence conservation and the structure of PRORP1 (17), the MRPP3 active-site region resides around D409, D478, D479 and D499 (Supplementary Figure S2). When comparing the structure of this region in Δ206-MRPP3 (Figure 2A) and the homologous PRORP1 (Figure 2B), it becomes clear that the Δ206-MRPP3 active site is distinctly different from that of PRORP1. Specifically, in PRORP1, D399^P^, D474^P^, D475^P^ and D493^P^ coordinate two magnesium or manganese ions (where ^P^ denotes PRORP1 numbering). Furthermore, N492^P^, which precedes the active-site residue D493^P^ in PRORP1, forms a bulge in the backbone and interacts with the backbone atoms of I519^P^, located in PRORP1 β6. This interaction is likely to stabilize the active-site area. In human Δ206-MRPP3, however, the equivalent residue to N492^P^ is R498, which occupies the metal-binding site, and forms a salt bridge with D478. At the same time, D499 is flipped out of the active site to hydrogen bond with the S569 of the lariat loop. In addition, the PRORP1 and Δ206-MRPP3 active-site areas differ around D478 and D479 as their relative positions are swapped in comparison to those of D475^P^ and D474^P^ in PRORP1 (Figure 2C). Here, D475^P^ and D474^P^ are located in the first turn of α18^P^ while, in contrast, the α18 in Δ206-MRPP3 starts first after the conserved P480, resulting in the observed swap.

The lack of metal ions bound in the active site was puzzling; therefore, in order to eliminate the possibility that this was due to the presence of metal-chelating citrate or malonate in the initial crystallization conditions, Δ206-MRPP3 was crystallized in alternative buffers (MES, HEPES) in the presence of excess Mg^2+^. However, no significant structural changes were observed in the active site (crystal 3; Table 1 and Supplementary Figure S3). Furthermore, we tested the functionality of the putative active-site residues in mature MRPP3 (residues 45–583) by site-directed mutagenesis (Figure 2D). The replacements D479N and D499N (D474^P^ and D493^P^) rendered the enzyme inactive, while D478N and D409N (D475^P^ and D399^P^) retained very low residual activity and partial activity, respectively.

Since the active sites of MRPP3 and PRORP1 only have a few key sequence differences, we wondered if MRPP3 could be made active independently of MRRP1/2 by changing the active-site area to be more similar to PRORP1. To this end, we mutated structurally relevant residues (single-point mutations: P480G, R498N, R498D, S569A; and one double mutant: P480G R498N). However, the mt-RNase P activity tests clearly showed that all of the mutants were able to perform the 5′-cleavage reaction in the presence of MRPP1/2 but not in their absence (Figure 2E). In a more drastic approach to recover MRPP1/2-independent activity, a MRPP3-PRORP1 chimera (MRPP3-MND^P^) was produced in which the complete MRPP3 metallonuclease domain was replaced by the PRORP1 metallonuclease domain. Cleavage tests showed that MRPP3-MND^P^ had very weak cleavage activity in the presence of MRPP1/2 and was still completely inactive on its own (Figure 2F).

### Structural comparison with *A. thaliana* PRORP1

**Figure 3.**
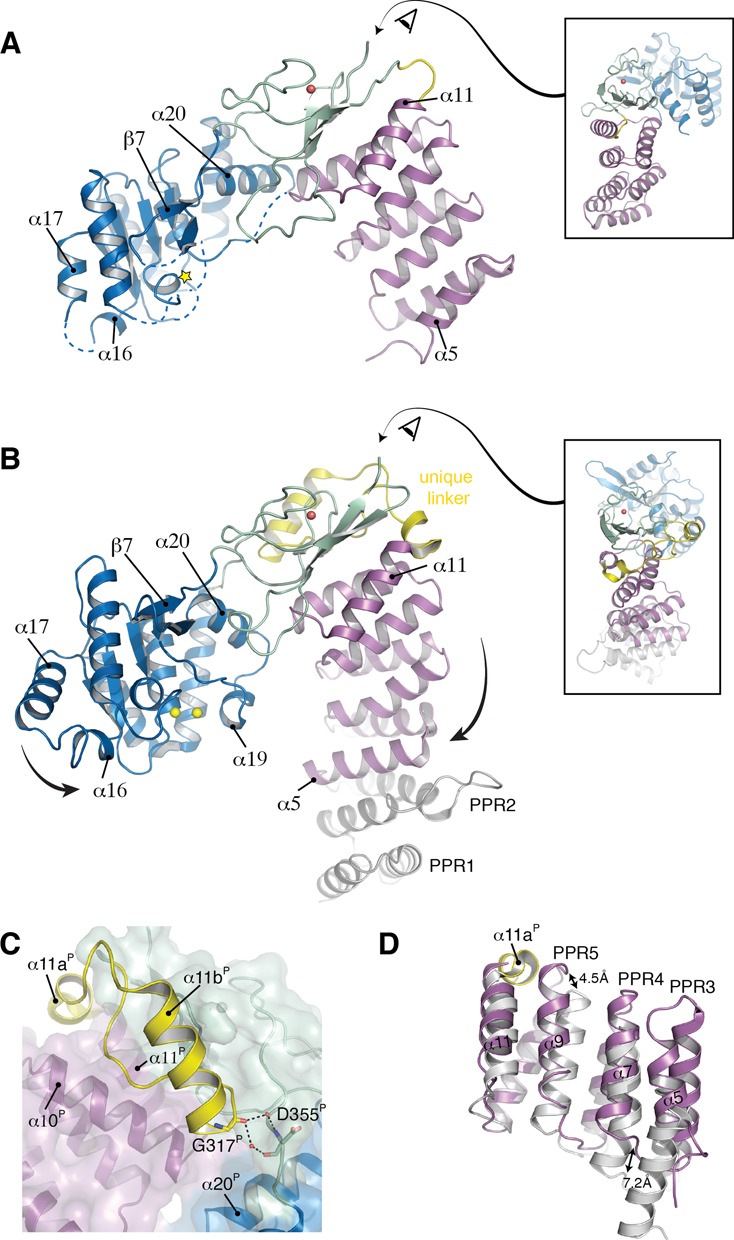
Structural comparison of MRPP3 and PRORP1. (**A**) Relaxed L-shape conformation of MRPP3 (1.80 Å resolution). [Box insert] The metallonuclease domain is translated out of a plane formed by the PPR and central domain. The eye indicates the viewing direction from the back. The color code is as in Figure 1. (**B**) V-shaped conformation of *A. thaliana* PRORP1 (PDB entry 4G24, 1.95 Å resolution, (17)). The plant unique linker harboring α11a^P^ and α11b^P^ is shown in yellow. The active-site manganese ions are shown in yellow spheres. Large arrows indicate the domain movements compared to MRPP3. [Box insert] All of the domains lie in the same plane. The eye indicates the viewing direction from the back. (**C**) Stabilization of the PRORP1 metallonuclease domain by helix α11b^P^. The water molecules are shown as red spheres. (**D**) The PRORP1 PPR domain. With ribbon presentation of the PPR domains of MRRP3 (purple) and PRORP1 (gray). Helix α11a^P^ of the PRORP1 unique linker (yellow surface) inserts in between α9^P^ and α11^P^, presumably stabilizing the orientation of the rigid PPR domain.

The overall structure of Δ206-MRPP3 (Figure 3A) is similar to that of *A. thaliana* PRORP1 (Figure 3B), with a PPR domain, a central domain and a metallonuclease domain. In addition, PRORP1 features a unique 30-residue-long linker region (Figures 3B+C and Supplementary Figure S2) that connects the PPR domain to the central domain. Although the overall folds of the individual domains of Δ206-MRPP3 and PRORP1 are similar (Supplementary Figure S4), there are two key differences.

The first key difference between Δ206-MRPP3 and PRORP1 is that the individual domains are oriented differently. Δ206-MRPP3 adopts a relaxed, twisted, L-shaped conformation with the metallonuclease domain being moved out of the plane formed by the PPR and the central domains (Figure 3A). In PRORP1, the PPR and metallonuclease domains are located closer together, forming a V-shape with all domains lying on one plane (Figure 3B). Taking the central domain as a references point, the Δ206-MRPP3 metallonuclease domain is rotated out by ∼40° compared to PRORP1 resulting in a 25 Å translational difference in the metallonuclease domain position. In addition, the PPR domain of Δ206-MRPP3 is located further out by ∼4.5 Å and 7 Å for α9 and α7, respectively (Figure 3D).

The second key difference is the organization of the lariat loop. In Δ206-MRPP3, the lariat loop forms an extended hydrogen bond network between the PPR and metallonuclease domain (Figure 1E). In contrast, the PRORP1 lariat loop is four residues shorter and adopts a horseshoe-like bend, in which the internal hydrogen bond network is concentrated within a hook that forms at the tip of the lariat loop (Supplementary Figure S5). Moreover, also in vast contrast to Δ206-MRPP3, there is only a single hydrogen bond with the PPR domain and a salt bridge with R518^P^, which is located in β6 of the metallonuclease domain. Furthermore, the lariat loop regions of Δ206-MRPP3 and PRORP1 are oriented at ∼90° to each other (Supplementary Figure S4B). Interestingly, the lariat loop sequence is strongly conserved in higher vertebrates (Supplementary Figure S2). To test whether removing the lariat loop from MRPP3 would result in MRPP1/2-independent activity, we replaced the MRPP3 lariat loop with a short linker (GSG). However, yet again, the MRPP3 mutant was only active in the presence of MRPP1/2 (Supplementary Figure S5C).

## DISCUSSION

Δ206-MRPP3 has the same overall structure as its plant homolog PRORP1; however, there are significant structural differences between the two proteins. The most striking difference is that the active site of Δ206-MRPP3 is in a non-productive conformation, in which no divalent metal ions are present. Furthermore, D499, which was shown to be essential for enzymatic activity, is rotated away while the preceding R498 occupies the proposed metal-binding site. Despite multiple attempts, it was not possible to load divalent metal ions into the active-site area. This supports the conclusion that the presence of a non-productive active site is physiologically authentic and not a crystallization artifact.

The non-productive active site observed in our MRPP3 structure raised the question as to whether MRPP3 is really the metallonuclease that is responsible for the endonucleolytic activity of mt-RNase P. To test this, we mutated the proposed metal-binding aspartic acid residues, which resulted in partial to complete inhibition of the enzymatic activity. This indicates that a proper metal-binding site, probably very similar to the PRORP1 active site, indeed must form in MRPP3 in order to perform the cleavage reaction. Similar active-site mutants have been created for PRORP family members in plants (13,15,17,29), but not for human MRPP3. Thereby, we experimentally confirmed that MRPP3 is the metallonuclease performing the endonucleolytic cleavage catalyzed by human mt-RNase P.

Sequence and structural comparisons of human MRPP3 and *A. thaliana* PRORP1 show only a few differences around the active-site area. Therefore, we anticipated that we could rebuild the MRPP3 active-site area following the architecture of *A. thaliana* PRORP1. This included replacing individual residues up to replacing the whole metallonuclease domain. However, we were not able to generate a human MRPP3 variant that was enzymatically active as a single protein. Hence, MRPP3 activity without MRPP1/2 does not hinge on just a few key sequence differences in the active site—or even differences in the metallonuclease domain as a whole—but on larger differences in the overall structure. We propose that MRPP3 is kept in a non-productive conformation by a dual-control mechanism, namely by the presence of a non-productive active site and by the orientation of the individual domains toward each other. However, structural information about MRPP1/2/3 in complex with the pre-tRNA substrate is required to fully elucidate how MRPP3 is transformed into a productive state in the tertiary complex.

An overall structural comparison between human Δ206-MRPP3 and *A. thaliana* PRORP1 points toward large differences in the localization of the individual domains. While Δ206-MRPP3 has a relaxed, twisted, L-shaped conformation, PRORP1 adopts a compact, V-shaped conformation in which all domains are located in one plane (Figure 3B). In PRORP1, the central domain is connected to the metallonuclease domain by a plant-specific, rigid, triple-proline motif (534-PPP-536 in PRORP1), thereby restricting the movement of the metallonuclease domain. In addition, the C-terminal end of the plant-unique helix α11b^P^ orients helices α12^P^ and α20^P^ (Figure 3C), leading to the stabilization of an active conformation of the PRORP1 active site. Furthermore, the PRORP1 PPR domain is oriented and stabilized by the plant-unique helix α11a^P^, which is inserted like a wedge on the outer face of the protein, in between α9^P^ and α11^P^ of the PPR domain (Figure 3D). It is clear that several plant-unique features of PRORP1 help orient the domains to be in one plane and stabilize the active site; we suggest that PRORP1 is preset in an active catalytic state.

In contrast to PRORP1, MRPP3 needs to switch from a non-productive to a productive conformation for catalysis to be possible. This conformational switch likely includes large domain movements, a reshaping of the lariat loop and a re-formation of the active site into a productive, catalytic state with bound active-site metals. As MRPP3 is only active in the presence of MRPP1/2 we propose that these large rearrangements are stabilized by MRPP1/2 and possibly also the pre-mt-tRNA substrate through an induced-fit mechanism. Thereby, our initial hypothesis of a structural latch regulating MRPP3's activity is supported, although the likely very large rearrangements necessary for nuclease activity were unexpected. Clearly, the structural data presented here show why MRPP3 is unable to function on its own, while a full molecular understanding of the regulation of mt-RNase P activity will require the structural determination of MRPP1/2/3 in a complex with its tRNA substrate.

The catalytically inactive construct of MRPP3, Δ206-MRPP3, which we have crystallized, lacks the N-terminal flexible regions and PPR motifs 1 and 2 raising the question if the absence of this region can influence the overall domain and active-site conformation. The functional role of the N-terminal flexible region remains unclear since its partial removal has no effect on tRNA processing (Supplementary Figure S1). Unfortunately, we could not achieve soluble expression of MRPP3 variants lacking the complete N-terminal flexible region while keeping all five PPR domains intact. However, similar experiments performed on *A. thaliana* PRORP1 showed that the enzymatic activity was reduced or abolished upon removal of the N-terminal flexible region and PPR motifs, respectively (19). Since the PRORP1 PPR motifs recognize the TΨC loop of the pre-tRNA (19), a similar role in substrate recognition can be assumed for MRPP3's PPR domain. Notably, the missing PPR1–2 motifs are >30 Å away from the metallonuclease domain on the other end of the PPR domain. Therefore, it is reasonable to assume that their absence will not influence the overall domain arrangement or the active site's conformation.

Given the role of mt-RNase P in the early stages of mitochondrial RNA processing (2–5) and the predicted requirement of m^1^A9 or m^1^G9 methylation for proper cloverleaf folding of the majority of the human mitochondrial tRNAs (7,10), it is tempting to speculate that the requirement of a ternary complex of MRPP1/2/3 is in fact a control mechanism in place to ensure that processed tRNAs will be folding competent after their release from the initial polycistronic transcripts.

## ACCESSION NUMBERS

The atomic coordinates and structure factors for MRPP3 crystals 2 and 3 have been deposited in the Protein Data Bank with the accession numbers 4XGL and 4XGM, respectively.

## SUPPLEMENTARY DATA

Supplementary Data are available at NAR Online.

SUPPLEMENTARY DATA
