# Supplementary material for: Structure of the nuclease subunit of human mitochondrial RNase P
Source: Nucleic Acids Res. 2015 May 7;43(11):5664–72. doi: 10.1093/nar/gkv481 (PMC4477676; doi:10.1093/nar/gkv481)
Supplement: SUPPLEMENTARY DATA [file supp_43_11_5664__index.html]

Structure of the nuclease subunit of human mitochondrial RNase P — SUPPLEMENTARY DATA 

# Structure of the nuclease subunit of human mitochondrial RNase P

## SUPPLEMENTARY DATA

- SUPPLEMENTARY DATA
