# Supplementary material for: Structure of the nuclease subunit of human mitochondrial RNase P
Source: Nucleic Acids Res. 2015 May 7;43(11):5664–72. doi: 10.1093/nar/gkv481 (PMC4477676; doi:10.1093/nar/gkv481)
Supplement: SUPPLEMENTARY DATA [file supp_gkv481_nar-00939-h-2015-File005.pdf]

SUPPLEMENTARY INFORMATION

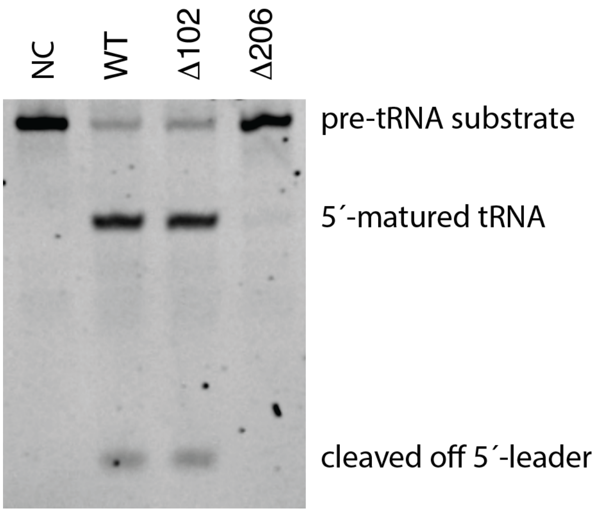

Supplementary Figure S1. Mt-RNase P assay of three N-terminal MRPP3 truncates showing the substrate and the two cleavage products. Reactions contain 40 nM pre-mt-$tRNA^{Tyr}$, 400 nM MRPP1/2 and 200 nM MRPP3 or its variants and were resolved on 7M urea 8% polyacrylamide gel. NC is the negative control. For simplicity and since the information content is the same, for all other presented mt-RNase P cleavage tests, cropped gel images are shown.

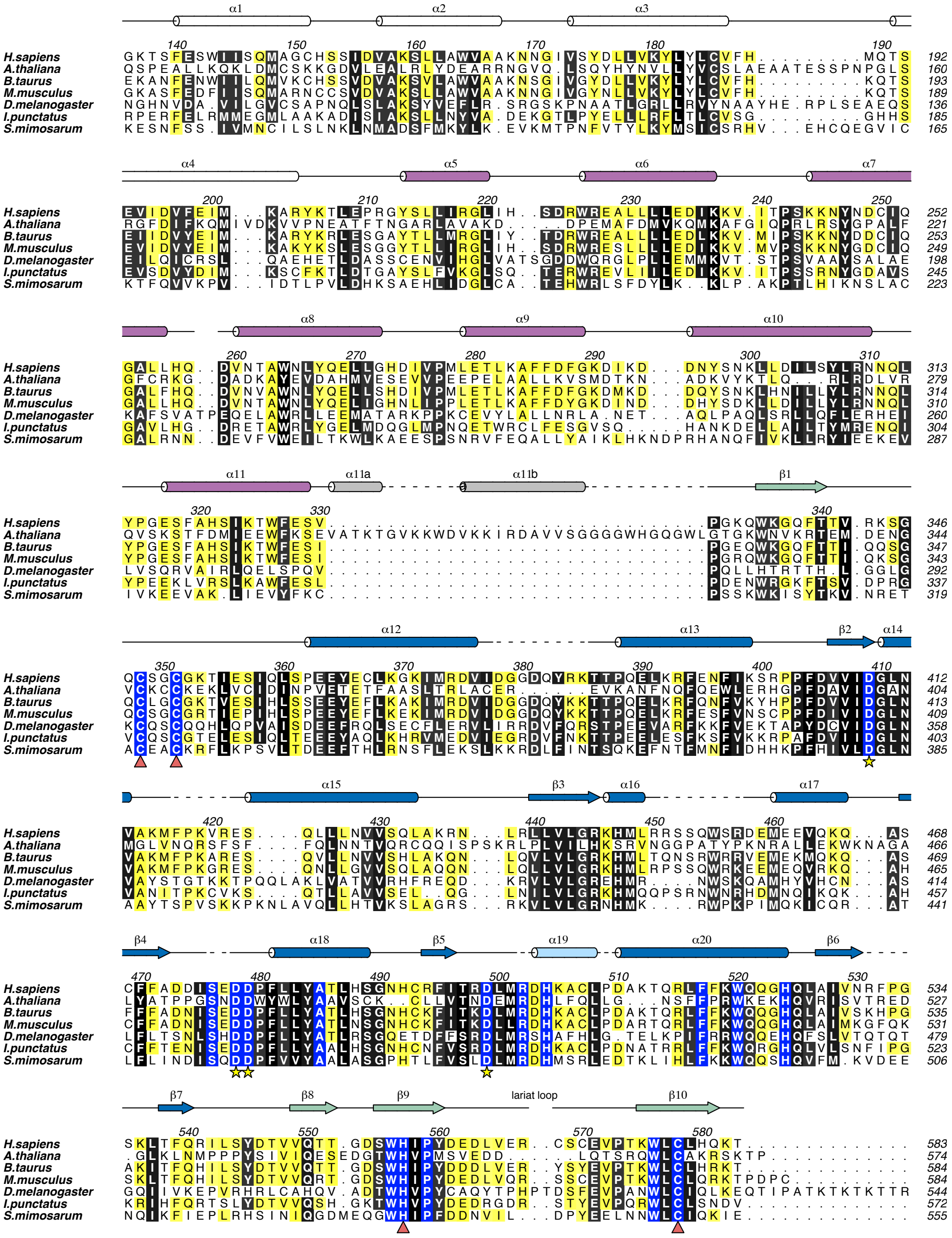

Supplementary Figure S2. Sequence alignment of MRPP3 homologs. The alignment was performed on 20 sequences, of which seven representatives are shown. The alignment of *H. sapiens* MRPP3 and *A. thaliana* PRORP1 is based on the three-dimensional structures. Residues which are similar between >50% and >75% of the 20 species are highlighted in yellow and gray scale, respectively. Strictly conserved residues are highlighted in blue. Secondary structure elements are shown for human MRPP3 with PPR domain in purple, central domain in green and the metallonuclease domain in blue. Elements not visible in the electron density are colored in pale and dashed lines. The locations of helices α1 to α4 (white) are based on secondary structure predictions. Helices α11a and α11b are of *A. thaliana* PRORP1 (1). Zinc binding and active site residues are marked by red triangles and yellow stars, respectively. The alignment was edited using ALINE (2).

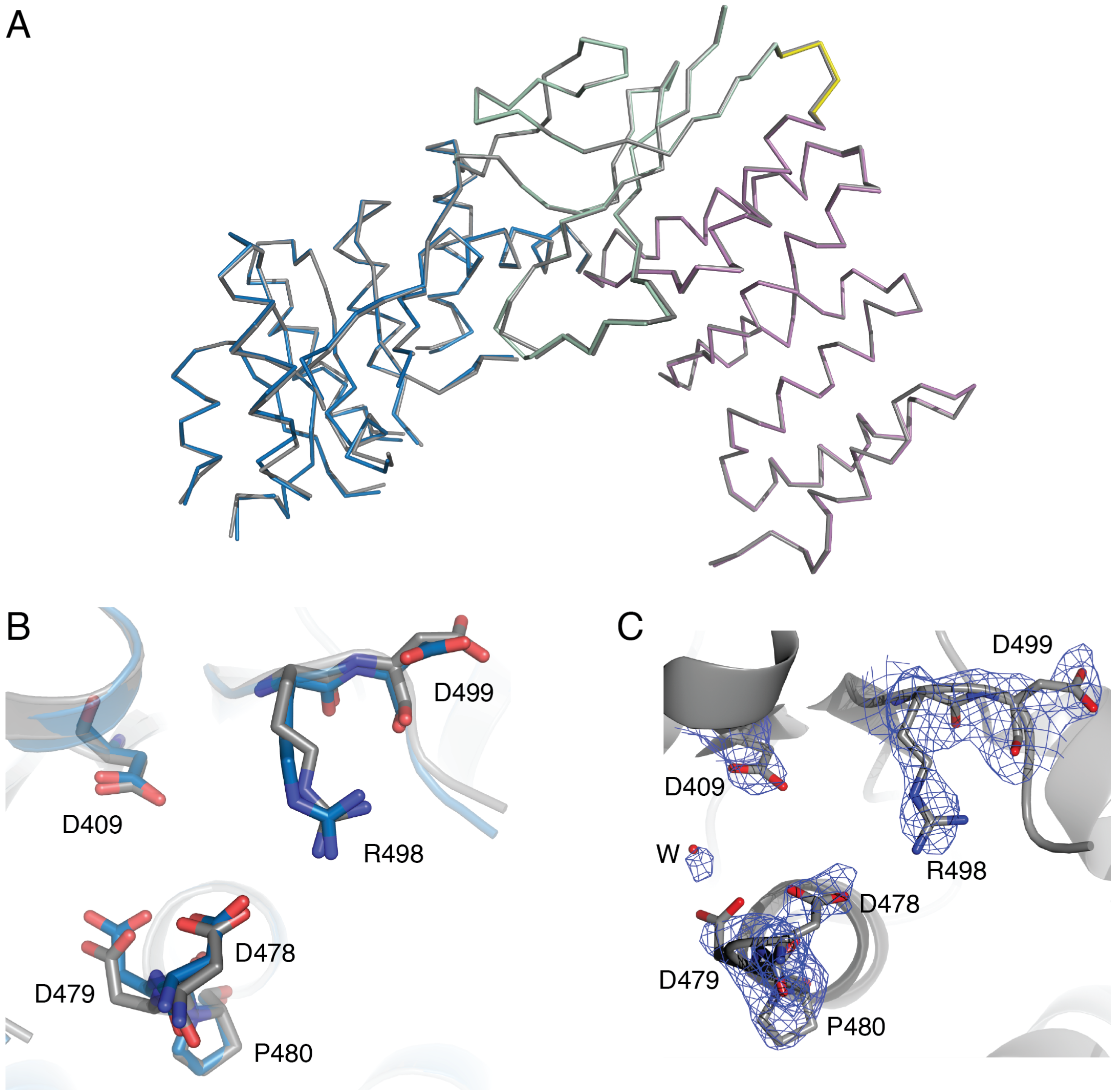

Supplementary Figure S3. Comparison of structural elements of MRRP3 crystal 2 (1.80 Å resolution) and crystal 3 (1.98 Å resolution). (**A**) Cα trace of the superposed MRPP3 structures. The structure of crystal 2 (citrate buffer pH 6.0) is colored as in Figure 1. The structure of crystal 3 (HEPES buffer pH 7.0) is colored in gray. The r.m.s.d. value of the superposition is 0.18 Å. Small structural changes are observed at the inner face of the metallonuclease domain. (**B**) The active site architectures observed in crystal 2 and 3. Active site residues are shown in sticks with crystal 2 in blue and crystal 3 in grey, respectively. (**C**) Electron density of the active site area in crystal 3. W indicates a water molecule. The 2FoFc map is shown at 0.9 σ.

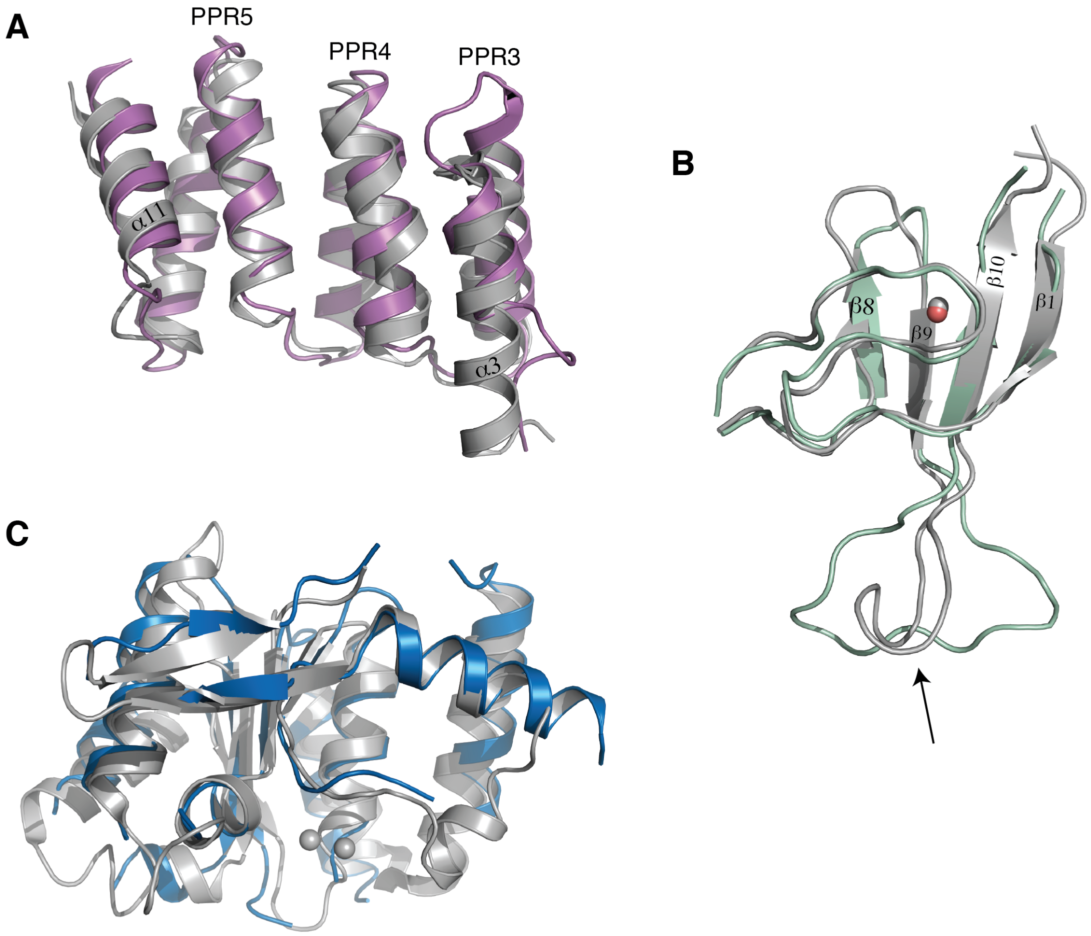

Supplementary Figure S4. Structural superposition of the individual MRPP3 and PRORP1 domains. MRPP3 (1.80 Å resolution) is shown in colors and PRORP1 in gray (pdb entry 4G24, 1.95 Å resolution, (1)). (**A**) Superposition of the PPR domains. PPR3-5 and α11 superpose with an r.m.s.d. value of 2.02 Å. For clarity, PPR1-2 of PRORP1 are not shown. The arrangement of PPR4, 5 and α11 is considerably similar, but PPR3 deviates significantly. (**B**) Superposition of the central domain. The lariat loop was excluded from the superposition procedure. The majority of the central domain is structurally conserved between MRPP3 and PRORP1 (r.m.s.d. value of 0.85 Å). The largest difference is observed for the conformation of the lariat loop (marked by an arrow). (**C**) Superposition of the metallonuclease domain (r.m.s.d. value of 2.45 Å). The two active site manganese ions of PRORP1 are shown in grey spheres.

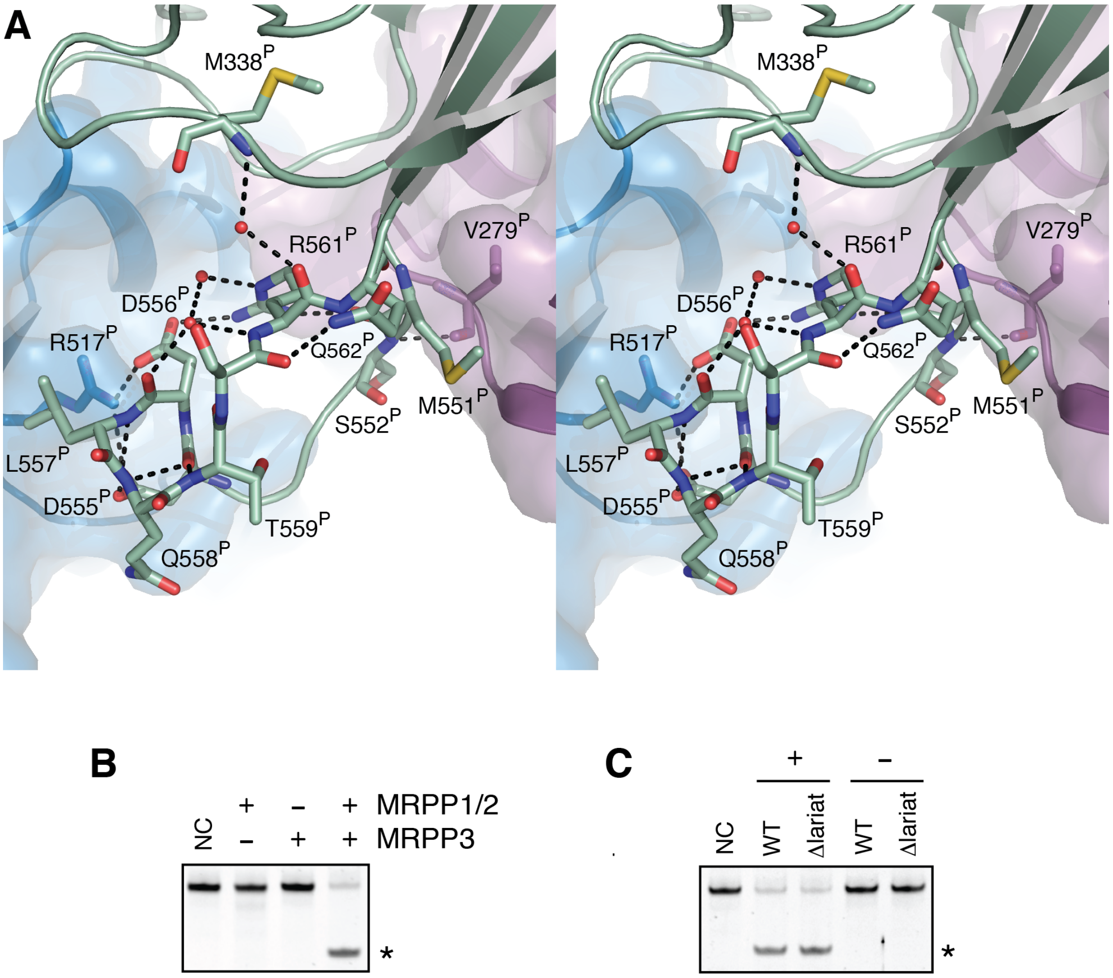

Supplementary Figure S5. Organization of the lariat loop. (**A**) The PPR domain, central domain and metallonuclease domains of PRORP1 (pdb entry 4G24, 1.95 Å resolution, (1)) are shown in purple, green and blue, respectively. Water molecules are shown in red spheres and interacting residues are shown in sticks. All hydrogen bonds have a distance less than 3.2 Å. (**B**) All three mt-RNase P components (MRPP1/2/3) are required to obtain mt-RNase P activity. Reactions contain 40 nM pre-mt-tRNA^{Tyr}, 400 nM MRPP1/2 and 200 nM MRPP3 and were resolved on 7M urea 8% polyacrylamide gels. NC is the negative control. An asterisk marks the cleavage product. (**C**) mt-RNase P activity of a mutant lacking the lariat loop (Δlariat). Activity was tested in the presence (+) and absence (-) of MRPP1/2.

SUPPLEMENTARY REFERENCES

1. Howard, M.J., Lim, W.H., Fierke, C.A. and Koutmos, M. (2012) Mitochondrial ribonuclease P structure provides insight into the evolution of catalytic strategies for precursor-tRNA 5' processing. *Proc Natl Acad Sci USA*, **109**, 16149-16154.
2. Bond, C.S. and Schuttelkopf, A.W. (2009) ALINE: a WYSIWYG protein-sequence alignment editor for publication-quality alignments. *Acta Crystallogr D Biol Crystallogr.*, **65**, 510-512.
